# Differential, Stage Dependent Detection of Peptidylarginine Deiminases and Protein Deimination in Lewy Body Diseases—Findings from a Pilot Study

**DOI:** 10.3390/ijms232113117

**Published:** 2022-10-28

**Authors:** Audrey Mercer, Zane Jaunmuktane, Mariya Hristova, Sigrun Lange

**Affiliations:** 1 Department of Pharmacology, UCL School of Pharmacy, London WC1N 1AX, UK; 2 Department of Clinical and Movement Neurosciences, Queen Square Brain Bank for Neurological Disorders, UCL Queen Square Institute of Neurology, London WC1N 3BG, UK; 3 Perinatal Brain Repair Group, Department of Neonatology, UCL Institute for Women’s Health, London WC1E 6HU, UK; 4 Tissue Architecture and Regeneration Research Group, School of Life Sciences, University of Westminster, London W1W 6XH, UK

**Keywords:** peptidylarginine deiminase, deimination/citrullination, post-translational modification, histone H3, neurodegeneration, Parkinson’s disease, Lewy body disease, brain

## Abstract

Over 10 million people worldwide live with Parkinson’s disease (PD) and 4% of affected people are diagnosed before the age of 50. Research on early PD-related pathways is therefore of considerable importance. Peptidylarginine deiminases (PADs) are a family of calcium-activated enzymes that, through post-translational deimination of arginine to citrulline, contribute to changes in protein function, including in pathological processes. Recent studies have highlighted roles for PADs in a range of neurological disorders including PD, but overall, investigations on PADs in Lewy body disease (LBD), including PD, are still scarce. Hence, the current pilot study aimed at performing an immunohistochemistry screen of post-mortem human brain sections from Braak stages 4-6 from PD patients, as well as patients with incidental LBD (ILBD). We assessed differences in PAD isozyme detection (assessing all five PADs), in total protein deimination/citrullination and histone H3 deimination—which is an indicator of epigenetic changes and extracellular trap formation (ETosis), which can elicit immune responses and has involvement in pathogenic conditions. The findings of our pilot study indicate that PADs and deimination are increased in cingulate cortex and hippocampus, particularly in earlier stages of the disease. PAD2 and PAD3 were the most strongly upregulated PAD isozymes, with some elevation also observed for PAD1, while PAD4 and PAD6 increase was less marked in PD brains. Total protein deimination and histone H3 deimination were furthermore increased in PD brains, with a considerable increase at earlier Braak stages, compared with controls. Our findings point to a significant contribution of PADs, which may further aid early disease biomarker discovery, in PD and other LBDs.

## 1. Introduction

More than 10 million people worldwide live with Parkinson’s disease (PD), the second most common age-related neurodegenerative disorder, and 4% of affected people are diagnosed before the age of 50. With no current cure for PD the identification of early PD-related pathways, that can serve as novel drug targets and aid early diagnosis, is of pivotal importance. A combination of clinical and post-mortem human patient samples, together with the use of PD animal models, may offer promising avenues for assessment of novel molecular pathways, and facilitate the identification of candidate pharmacological lead compounds for therapeutic intervention. The use of post-mortem human brain samples from patients with PD, at various disease stages as determined by Braak et al. [1], can aid initial identification of changes in molecular pathways and histopathological changes, including early onset ones.

Peptidylarginine deiminases (PADs) are a family of five calcium-activated enzymes (PAD1, PAD2, PAD3, PAD4 and PAD6) that, through post-translational deimination/citrullination of arginine to citrulline, contribute to changes in protein function, including that of cytoskeletal, structural and mitochondrial proteins, intermediate filaments and histones, therefore also affecting epigenetic regulation [2,3,4,5,6]. PAD-mediated protein deimination is detected in a range of neurodegenerative, inflammatory and other chronic systemic diseases, with evidence for differences in PAD isozyme specific roles these diseases [7,8,9,10,11,12,13]. Importantly, a recent study by our group identified elevated PAD levels and deimination in brains of an early stage PD rat model [14]. In addition, we identified that circulatory deimination signatures in plasma and plasma-derived extracellular vesicles (EVs) were elevated and modified [14]. As PADs are a modulator of EV signatures [15,16,17,18], changes in PAD expression may influence EV mediated roles in cell communication and pathological processes, including in neurological disorders, where increasing research evidence highlights the role of EVs [19,20], including for misfolded protein transcellular transmission [21].

Importantly, the role for PADs as drug targets in early PD may be of considerable interest as in several CNS injury animal models, an effective neuroprotective role for pharmacological PAD-inhibition has been described [22,23]. This has included reduced neuroinflammation, cell death and histone H3 deimination, which is indicative of gene regulatory changes and formation of extracellular traps (ETosis), which contain DNA, histones and cellular proteins and form part of pathogenic responses, while they can also contribute to autoinflammatory injury [22,23]. Studies from other groups on PD post-mortem human brains have highlighted altered deimination in PD [24,25], including increased histone H3 deimination in X-linked Dystonia Parkinsonism post-mortem prefrontal cortex [26]. In vitro PD models have also indicated that pharmacological PAD inhibition reduces inflammatory responses [26]. These previous findings formed the basis of the current pilot study as overall; the literature on PAD-mediated processes in Lewy body disease (LBD) is still scarce.

The current study aimed at carrying out a pilot screen using post-mortem human PD brain samples of Braak stages 4–6 and incidental Lewy body disease (ILBD), which may represent pre-clinical PD [27], to further the understanding of PAD isozymes and protein deimination at various stages of LBD progression.

## 2. Results

### 2.1. Immunohistochemical Detection of PAD Isozymes, in Anterior Cingulate Cortex and Hippocampus of Post-Mortem Human PD Brains

**Figure 1:**
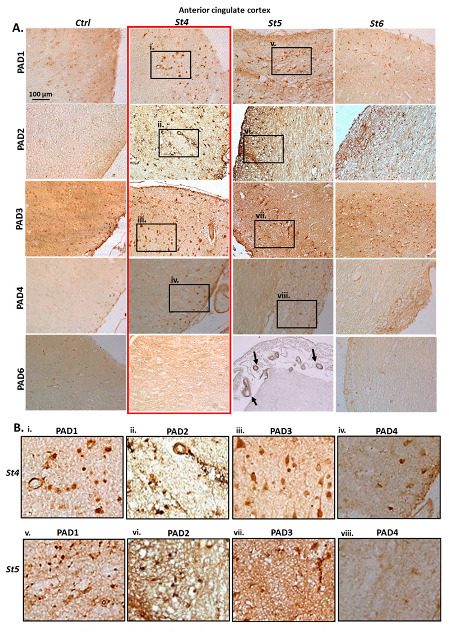
PAD isoform detection in anterior cingulate cortex tissue sections showing immunohistochemical staining of PAD isozymes (PADs 1, 2, 3, 4 and 6), in human post-mortem PD brains: (**A**) Brains at Braak stages 4, 5, 6, compared with control brains; Highest staining for PADs is observed at earlier Braak stages, notable at stage 4 (as highlighted by the red rectangle), Scale bar represents 100 μm; black rectangles (i–viii) highlight areas that are further magnified in B; Black arrows point to positively stained brain vasculature. (**B**) Magnified images are shown from the regions outlined by black rectangles in A (i–viii), and highlight the observed increase of positive staining at St4 for PADs 1–4; while some strong positive staining was also observed at stage 5 for PAD1, PAD2 and PAD3.

**Figure 2:**
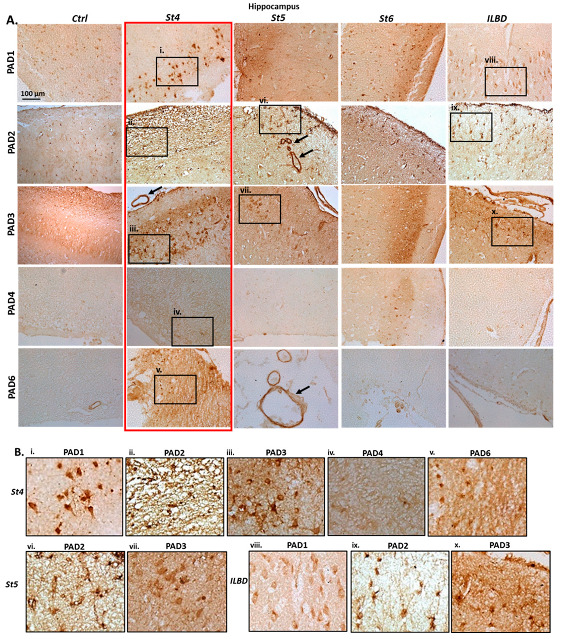
PAD isozyme (PADs 1–6) detection by immunohistochemistry in hippocampal tissue sections of post-mortem human PD brains. (**A**) Braak stages 4, 5, 6 and ILBD, Highest PAD levels are observed at earlier PD stages, notable at stage 4 (as highlighted by the red rectangle). Detection of PADs 1–3 is also notable in brain with ILBD. Scale bar represents 100 μm. Black arrows point to positively stained brain vasculature. The black boxes (i–x) indicate areas that are further magnified in B. (**B**) (i–x): strong detection of PAD1, PAD2 and PAD3, as well as notable detection of PAD6 at Braak stage 4 (St4) is observed. PAD2 and PAD3 positive detection is clear at Braak stage 5 (St5). In brains with ILBD, PAD1 was clearly detectable, and both PAD2 and PAD3 showed strong positive staining.

PAD isozymes’ levels were assessed and detected in anterior cingulate cortex and hippocampus of post-mortem brains. Control age matched brains were used for comparison with PD brains at Braak stages 4, 5 and 6. Staining for anterior cingulate cortex is shown in Figure 1. Hippocampus was assessed at the same Braak stages and in addition in brain with incidental Lewy body disease (ILBD), represenatative of pre-clinical stages of PD (Figure 2). Representative images for staining of PAD isozymes is shown for anterior cingulate cortex in Figure 1 and for hippocampus in Figure 2; highlighting the strong PAD detection at Braak stage 4, as indicated by the red rectangles. In Figure 1, some faint positive is seen for PAD1 in anterior cingulate cortex of control brains (Ctrl), with a sharp increase of positive staining at Braak Stage 4 (St4), with still strong detection at Braak stage 5 (St5), but lower detection, similar to control brain tissue, for Braak stage 6 (St6). For PAD2, negligible detection was observed in control brains (Ctrl), very high PAD2 detection at Braak stage 4 (St4), with high detection also at Braak stage 5 (St5), and lower positive detection at Braak stage 6 (St6). PAD3 detection was strong in control brains (Ctrl) compared with the other PAD isozymes, but was markedly increased in Braak stage 4 brains (St4), with high PAD3 detection also in Braak stage 5 brains (St5), but lower in Braak stage 6 brains (St6). PAD4 detection was negligible in control brains (Ctrl), somewhat elevated in Braak stage 4 brains (St4), but very low positive PAD4 detection was observed in brains at Braak stages 5 (St5) and 6 (St6). PAD6 was detected a low levels in control brains (Ctrl), with some increase at Braak stage 4 (St4), and positive vascular staining at Braak stage 5 (St5; black arrows), while detection was low at Braak stage 6 (St6).

As shown in Figure 2, some positive PAD1 staining was observed in hippocampus of control brains (Ctrl), with a very strong detection at Braak stage 4 (St4), but lower detection at Braak stage 5 (St5) and 6 (St6), while some clear positive neuronal detection was also observed in hippocampus with incidental Lewy body disease (ILBD), which was somewhat higher than in control brains. PAD2 detection showed low positive in control hippocampus (Ctrl), was clearly elevated at Braak stage 4 (St4), and also in Braak stage 5 (St5), where strong positive staining was also observed in the brain vasculature (black arrows). PAD2 detection was also clear at Braak stage 6 (St6), including strong positive labelling in astrocytes. PAD2 detection was also strong in hippocampus of brain with ILBD. PAD3 staining was detectable in hippocampus of control brain (Ctrl), was very strongly elevated at Braak stage 4 (St4), and observed at lower levels, but still clearly positive at Braak stage 5 (St5). PAD3 detection was more diffuse positive at Braak stage 6 (St6), and strong in hippocampus with ILBD. PAD4 detection was negligible in control brain (Ctrl), showed some low level of elevation at Braak stage 4 (St4), was negligible at Braak stage 5 (St5) and some occasional positive staining was observed at Braak stage 6 (St6). Hippocampus with ILBD showed negligible for PAD4 staining. PAD6 staining was negligible in control brain, except in some vasculature, but was strongly elevated in Braak stage 4 brain (St4), with strong labelling in the vasculature in Braak stage 5 brain (St5; black arrow), as well as some occasional positive reactivity in the vasculature in Braak stage 6 brain (St6), but PAD6 staining was low to moderate in hippocampus with ILBD.

### 2.2. Immunohistochemical Detection of Histone H3 Deimination and Pan-Deimination in Anterior Cingulate Cortex and Hippocampus of Post-Mortem Human PD Brain Sections

**Figure 3:**
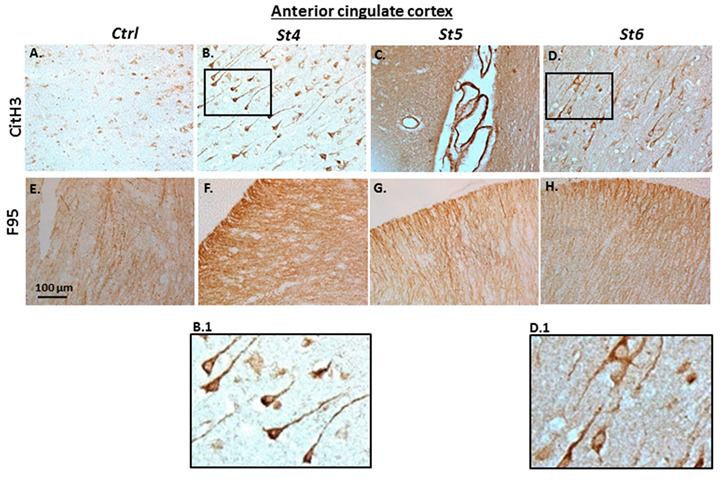
(**A**–**H**) Post-mortem human brain tissue sections of anterior cingulate cortex, showing immunohistochemical detection of histone H3 deimination (CitH3) and pan-deimination (F95). Braak stages 4, 5 and 6 are shown, in comparison with control brain (Ctrl). Scale bar represents 100 μm. The black rectangles in (**B**,**D**). are magnified in B.1 and D.1, respectively.

**Figure 4:**
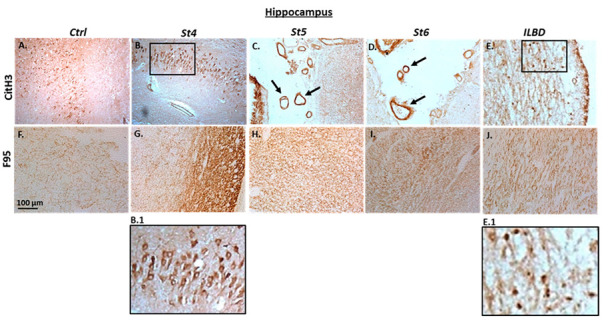
(**A**–**J**) Post-mortem human brain tissue sections of hippocampus, showing neuronal cytoplasmic immunohistochemical detection of histone H3 deimination (CitH3) and pan-deimination (F95). PD brains at Braak stages 4, 5 and 6, as well as hippocampus with incidental Lewy body disease (ILBD) are shown, alongside control hippocampus (Ctrl). Scale bar represents 100 μm. The black rectangles in (**B**,**E**) are magnified in B.1 and E.1, respectively; black arrows point at positively stained brain vasculature.

Immunohistochemical staining for deiminated histone H3 (CitH3) and for pan-deimination, as detected by the pan-citrulline F95 antibody [28], is shown for anterior cingulate cortex (Figure 3) and for hippocampus (Figure 4). Control tissue is shown in comparison with PD brains at Braak stages 4, 5 and 6, in addition to hippocampus with ILBD (representative of pre-clinical PD). In anterior cingulate cortex, CitH3 staining was low in control brain tissue (Figure 3A), very strongly positive neuronal cytoplasmic staining was observed at Braak stage 4 (Figure 3B), and a lower, albeit strong positive staining at Braak stages 5, particularly in the brain vasculature (Figure 3C, arrows) and strong neuronal staining at Braak stage 6 (Figure 3D). A clear positive staining was observed for pan-deimination (F95) in anterior cingulate cortex of control brain (Figure 3E), albeit lower than in the PD brains (Figs 3F-H). F95 staining was highest in anterior cingulate cortex at Braak stage 4 (Figure 3F), and still strong, albeit lower at Braak stages 5 and 6 (Figure 3G,H).

A similar pattern was observed in hippocampus (Figure 4), where CitH3 detection was strongest in PD brains at Braak stage 4 showing strong neuronal cytoplasmic staining (St4, Figure 4B), while clear positive staining was also observed in control brain (Figure 4A). Positive neuronal staining, as well as strong positive labelling in the brain vasculature was observed for CitH3 at Braak stages 5 and 6 (Figure 4C,D; arrows). Furthermore, a strong neuronal detection for CitH3 was observed in hippocampus with ILBD (Figure 4E). For pan-deimination detection, some positive F95 staining (possibly synaptic labelling in the neuropil) was observed in control hippocampus (Figure 4F), which was stronger at Braak stage 4 (Figure 4G) and clear positive staining was also seen in hippocampus of Braak stages 5 and 6 (Figure 4H,I), as well as strong positive staining in hippocampus with ILBD (Figure 4J).

A summary of immunohistochemical detection for PAD isozymes, pan-deimination and histone H3 deimination of post-mortem PD brains at the different Braak stages, and hippocampus with ILBD, is presented in Table 1, according to the staining intensity key shown in Supplementary Figure S1. Negative control brain sections, omitting the primary antibody, are furthermore shown in Supplementary Figure S2.

## 3. Discussion

The current pilot immunohistochemistry screen assessed human post-mortem brain tissue from Parkinson’s disease (PD) at Braak stages 4–6 and incidental Lewy body disease (ILBD; representative of pre-clinical PD [27]), highlighting elevated protein levels of specific PAD isozymes and increased deimination and histone H3 deimination in early stages of PD. This links in with recent findings of the authors, describing a novel PAD-related brain pathology, including elevated PADs and increased deimination and histone H3 deimination, in a rat model of pre-motor PD [14]. This included the identification of increased pan-deimination in the brain vasculature [14], which interestingly was also observed in the current study in some of the human post-mortem PD brain sections, in particular in hippocampus at Braak stage 5. Interestingly, a previous study on Alzheimer’s disease (AD) post-mortem human brain samples identified pan-deimination in proximity with small intraparenchymal blood vessels and in walls of extraparenchymal blood vessels [29]. It may therefore be postulated whether deimination-linked changes are also present in extraparenchymal blood vessels in other neurodegenerative disorders, including PD, although this will require further assessment. Studies by other groups have reported increased protein deimination in post-mortem PD brains, including in surviving dopamine neurones in the substantia nigra, although not specifically restricted to Lewy bodies, indicating alteration of PADs in PD [24]. Furthermore, mutated misfolded α-synuclein protein has been related to increased protein deimination [25]. Also, increased PAD2 and PAD4 levels, as well as H3 citrullination/deimination were identified in post-mortem prefrontal cortex of patients with X-linked Dystonia Parkinsonism [26]; albeit other PAD isoforms were not assessed in that study, contrary to our current study where all five PADs were assessed in post-mortem PD brains. Increased deimination has furthermore been reported in several acute brain injury models, including acute CNS injury, hypoxic ischaemic insult and traumatic brain injury [22,23,30,31].

Importantly, our current pilot screen of human post-mortem PD brain samples further indicates significant increase of selected PADs and protein deimination in anterior cingulate cortex and hippocampus, particularly in earlier stages of the disease. These two brain regions were chosen for this current study due to their involvement in Lewy body diseases [32,33]. Hippocampus was assessed for ILBD, which can represent pre-clinical PD, while both regions were assessed for PD, which is generally verified at Braak stages 3 to 4, hence PD brain sections were selected from Braak stage 4 to 6 to assess PAD and deimination staining in PD progression. Interestingly, in anterior cingulate cortex all PAD isozymes were elevated, compared with control brains, at Braak stage 4, with particularly elevated protein levels of PAD1, PAD2 and PAD3, while elevation of PAD4 was low, and some elevation was seen for PAD6. Similarly, in hippocampus PAD1, PAD2, PAD3 were most elevated in the PD brains, particularly at Braak stage 4, while increase in PAD4 was comparably small, and increase in PAD6 was mainly linked to the brain vasculature. These findings do indicate possible hitherto overlooked roles for PAD1 in neurodegeneration, and also emphasise the role for PAD3 in CNS pathology. PAD3 has indeed been linked to acute CNS inflammation and repair [22] and neuronal stem-ness [34], as well as to aggressive CNS tumours [17,18]. Generally, PAD2 is regarded as the dominant isozyme in brain [35], and for example linked to Alzheimer’s disease (AD) [7,8], prion disease [36] and amyotrophic lateral sclerosis (ALS) [13]. Both PAD2 and PAD4 have previously been assessed in relation to X-lined dystonia PD [26]. Our current findings from this pilot study indicate that PAD4 is not as increased as the other PADs in the PD brains, while this isozyme has for example been linked to multiple sclerosis (MS) [37] and also showed some elevated response in rat pre-motor PD model brains [14]. PAD6 was also less elevated than PAD1-3 in the PD brains in this study, but did though show some higher levels compared with control brains. A putative role for PAD6 in PD may be of some interest, as this isozyme has recently been linked to hypoxia responses in naked mole-rat brain [38], but otherwise not previously linked to neuronal injury and mainly been linked to developmental processes [39,40,41], while recently also suggested to have roles in some animal cancers [42]. Overall, there may be a need for more in depth investigations into the individual PAD isozymes in PD, as the identification of PAD isozyme specific roles in different neurodegenerative disorders may be of considerable importance.

In this study, histone H3 deimination was observed to be strongly increased in neurones of PD brains at Braak stage 4 and observed in the brain vasculature at later Braak stages, while CitH3 positive neurones were also observed in ILBD. Histone H3 deimination can be indicative of epigenetic regulation and also of extracellular trap formation (ETosis), which due to pathogenic responses and associated inflammatory function is linked to brain injury [43]. Interestingly in vitro pharmacological PAD inhibition in PD-derived fibroblasts showed that the pan-PAD-inhibitor Cl-amidine reduces histone H3 deimination and pro-inflammatory chemokine expression [26], while such PAD inhibition has been shown to disrupt neutrophil extracellular trap (NET) formation also in other disease models [44].

The observed increase in PAD isozymes and protein deimination in the current study in human PD post-mortem brains correlates with our recently published animal study identifying increased PAD levels and protein deimination in brains and plasma of pre-motor PD animal models, where furthermore we also found raised levels of plasma-extracellular vesicles (EVs) with a modified content of deiminated proteins [14]. Indeed, PD patients have been shown to have greater amounts of circulating EVs [45,46], while the role for EVs in early pre-motor PD stages still remains a relatively unexplored area. The link between elevated PAD expression and changes in EV regulation in neurodegenerative disorders, including PD may be of some interest. The plasma-EV citrullinome “fingerprint” in the pre-motor PD animal model was indeed related to neuro-degenerative and neuro-inflammatory KEGG pathways including “Parkinson’s Disease” “Alzheimer’s disease”, “Huntington’s disease”, “prion diseases”, “oxidative phosphorylation” and “metabolic pathways” [14]. This points to significant roles for deimination in neurodegenerative processes and may also indicate a link between brain-related changes linked to PADs, including isozyme specific ones, and systemic deimination signatures. As epigenetic mechanisms of PD are receiving increased attention, including post-translational modifications [14,47,48,49], deimination may be of considerable interest and requires further investigation. This is supported both by the current findings reported here, and previous findings from both animal models and human studies, pointing increasingly to a significant contribution of post-translational deimination in PD, which importantly may aid biomarker discovery at early disease stages. Previous in vitro studies by the authors furthermore identified increased protein deimination in human PD iPSC models of α-synyclein triplication [50]. Collectively these findings emphasise the putative role for selected PAD isozymes as important players in Lewy body disease.

Targeting PADs may be a promising avenue in PD therapeutics. In addition to in vitro results showing that pan-PAD-inhibitor Cl-amidine reduces histone H3 deimination and pro-inflammatory chemokine expression in PD-derived fibroblasts [26], animal studies of acute CNS injury have demonstrated major roles for PADs (highlighting PAD3) and significant neuroprotective effects using pharmacological pan-PAD inhibition [22,23]. PAD isozyme specific regulation and application of PAD isozyme specific inhibitors (PAD2, PAD3 and PAD4 specific ones) have also been applied in CNS injury models in vitro (brain cancer) [17,18]. Furthermore, significant roles for PADs have been described in modulating EV disease-specific signatures, including via pharmacological PAD-inhibitors in a number of chronic pathologies, including in the CNS [17,18]. Critically, EVs are currently gaining increasing attention in relation to neurodegenerative disease, including PD, due to their potential use as non-invasive markers via identification of specific EV-cargo (“EV-fingerprint”), while current knowledge on EVs in pre-motor PD and early stages of PD is still limited [14,51,52,53]. Hence, PAD-mediated effects (including isozyme specific ones) may be of interest on such signatures, particularly as in neurodegenerative diseases, both neurotoxic and neuroprotective roles via distribution of EV-mediated cargo, including misfolded proteins, have been implicated [54,55]. Accumulative evidence therefore supports pathological roles of PADs in PD brains and indicates a possible link to PAD-mediated effects on circulatory EV-signatures. This, in combination with effective roles of PAD-inhibitors in CNS repair, highlights selected PAD-mediated pathways as promising targets for novel therapeutic intervention in early PD and for disease monitoring.

It must be pointed out that an obvious limitation of our present pilot study is the use of only one patient per Braak stage. In addition, the current study focussed on two brain regions only, namely anterior cingulate cortex and hippocampus. Nonetheless, our pilot findings indicate that some PAD isozymes are notably elevated in PD at earlier Braak stages and in ILBD. Further assessment of PAD isozyme specific detection and the associated deiminated target proteins in the different brain regions involved in PD (the brain-region specific “citrullinome”), may furthermore be of interest, and has been for example been noted to differ in traumatic brain injury [30]. Importantly, it must also be considered that the various PAD isozymes have different preferences for target proteins [56], which may also contribute to changes in the PD-related citrullinome with disease progression, including in the various brain regions. Such isozyme-specific differences must also be considered in relation to variations in PAD mediated responses in different neurodegenerative diseases, which may allow for isozyme-targeted treatments for different diseases. While limited to immunohistochemistry analysis, the current pilot report lays foundation for further studies into PAD-mediated responses, including at earlier stages of Lewy body disease. Analysis of PADs and protein deimination in larger number of patients will furthermore be required to confirm the changes observed in this study, alongside investigations into other brain regions. Our findings support that deimination is a possible indicator for monitoring of disease progression, and highlights selected PADs as molecular targets in early stages of disease.

## 4. Materials and Methods

### 4.1. Human Post-Mortem Brain Sections

Paraffin embedded post-mortem human brain tissue sections from anterior cingulate cortex and hippocampus were obtained from the UCL Queen Square Brain Bank with written informed consent for tissue usage in research for all cases and ethical approval of the study in place. The histological diagnosis and Braak stage of the Parkinson’s disease were confirmed by a neuropathologist (ZJ). For the purpose of the current pilot study, only one patient per PD Braak stage and incidental Lewy body disease (ILBD), respectively, were assessed. Control brain sections were from an age matched individual. Table 2 provides a summary of the brain samples used.

### 4.2. Immunohistochemistry

Deparaffinised tissue sections (7 μm serial sections), from the anterior cingulate cortex at the level of the nucleus accumbens and the posterior hippocampus at the level of the lateral geniculate nucleus, were stained for the detection of the five PAD isozymes PAD1,2,3,4 and 6, for deiminated histone H3 (CitH3) and for pan-deimination (F95 pan-citrulline/deimination antibody; [28]), using methods described in previous studies [42,57]. In brief, the sections were first deparaffinised three times in xylene for 10 min, then immersed for 5 min in 100% isopropanol and rehydrated with incubation in ethanol (100, 90% and 70%) for 5 min each. Sections were incubated in water (dH_2_O) and then heated in citric acid buffer (pH 6.0) in the microwave for 12.5 min, at power 8 for antigen retrieval. Sections were left to cool to room temperature (RT), incubated in distilled water and then blocked in 5% goat serum (Sigma, St. Louis, MO, USA) in phosphate buffer (PB) for 1 h. Primary antibody incubation was carried out overnight at 4 °C in a humidified chamber, diluting the antibodies 1/100. The primary antibodies used in this study are listed in Table 3.

Following primary antibody incubation, sections were washed in 100 mM PB and then incubated in the secondary antibody solution for one hour at RT (anti-rabbit IgG or anti-mouse IgM biotinylated antibodies (Vector laboratories, Peterborough, UK; diluted 1/200). Sections were then first incubated with Avidin-Biotinylated peroxidase Complex (ABC, Vector Laboratories) for 1 h, at RT, then with diaminobenzidine/hydrogen peroxide (DAB) stain for 5 min at RT, and finally with Mayer’s haematoxylin (Sigma, Gillingham, UK) for background staining. Following the peroxidase staining, sections were dehydrated in alcohol (70%, 90%, 100%—5 min each), incubated in xylene, mounted onto slides using DEPEX (Sigma) and cover slipped. 20x digital images were captured using a Leica microscope and a Sony AVT-Horn 3CCD colour video camera (24 bit RGB, 760 × 570 pixel resolution). For an estimation of staining intensity, the following score was determined: 0 as no labelling; + as weak labelling; ++ for moderate labelling; and +++ for strong labelling; a representative staining scoring key is shown in Supplementary Figure S1.

## 5. Conclusions

This pilot immunohistochemistry study assessed peptidylarginine deiminase isozymes (PAD1, PAD2, PAD3, PAD4 and PAD6) and protein deimination in post-mortem human brains with incidental Lewy body disease and Parkinson’s disease (PD) at Braak stages 4–6. Our findings indicate an increase in PAD isozyme protein levels and protein deimination at earlier Braak stages, with PAD2 and PAD3 being the most strongly upregulated isozymes. PAD1 and PAD6 were also found to be increased compared with controls, while less increase was observed for PAD4. A strong increase in histone H3 deimination (CitH3) was observed, alongside increase in total protein deimination. This is the first study to assess all five PAD isozymes and deimination in post-mortem human PD brains at different Braak stages. Our findings highlight PAD isozymes as candidate early disease biomarkers in LBD, while further in depth studies are needed.

## Figures and Tables

**Table 1:** Summary of immunohistochemical detection of PAD isozymes, histone H3 deimination (CitH3) and pan-deimination (F95) in anterior cingulate cortex (ACC) and hippocampus (HIP) of post-mortem human PD brains at Braak stages 4-6 and incidental Lewy body disease (ILBD). Scoring is shown as 0,+,++ and +++, respectively, based on the scoring index in Supplementary Figure S1. 0 indicates negligible staining; + low positive, ++ medium positive, and +++ strong positive staining; na is indicated for ACC as ILBD was assessed in hippocampus only.

	PAD1		PAD2		PAD3		PAD4		PAD6		F95		CitH3	
Sample	ACC	HIP	ACC	HIP	ACC	HIP	ACC	HIP	ACC	HIP	ACC	HIP	ACC	HIP
Control	+	+	0	+	+	+	(+)	0	(+)	0	+	(+)	+	+
Braak Stage 4	+++	+++	+++	+++	+++	+++	++	+	+	+++	+++	+++	+++	+++
Braak Stage 5	++	+	++	+++	++	++	+	0	+	++	++	++	++	+++
Braak Stage 6	+	+	++	++	++	+	+	+	+	+	+	+	++	+
ILBD	na	++	na	++	na	++	na	0	na	+	na	++	na	++

**Table 2:** Summary of post-mortem PD and ILBD brain tissue, alongside age matched control, used in the current pilot study; na is indicated for ILBD as only hippocampus was assessed.

Brain Samples	N	Anterior Cingulate Cortex (ACC)	Hippocampus (Hip)	Sex	Age at Death
Control	1	v	v	F	86
PD Stage 4	1	v	v	M	76
PD Stage 5	1	v	v	M	81
PD Stage 6	1	v	v	M	87
ILBD	1	na	v	F	82

**Table 3:** Primary antibodies used for immunohistochemical detection in post-mortem human brain tissue samples.

Antibody	Cat No	Supplier
Anti-human PAD1	ab181762	Abcam, Cambridge, UK
Anti-human PAD2	ab50257	Abcam
Anti-human PAD3	ab50246	Abcam
Anti-human PAD4	ab50247	Abcam
Anti-human PAD6	PA5-72059	Thermo Fisher Scientific, Oxford, UK
Anti-histone H3 deimination (citrulline R2R8R17) antibody (CitH3)	ab5103	Abcam
Pan-citrulline/deimination F95 antibody	MABN328	Merck, Feltham, UK

## Data Availability

All data generated for this study is included in the published article and Supplementary Materials.

## Supplementary Materials

The following supporting information can be downloaded at: https://www.mdpi.com/article/10.3390/ijms232113117/s1.
