# Supplementary material for: Differential, Stage Dependent Detection of Peptidylarginine Deiminases and Protein Deimination in Lewy Body Diseases—Findings from a Pilot Study
Source: Int J Mol Sci. 2022 Oct 28;23(21):13117. doi: 10.3390/ijms232113117 (PMC9658624; doi:10.3390/ijms232113117)
Supplement: Supplementary file 1 [file ijms-23-13117-s001.zip › ijms-1984739 - supplementary.pdf]

International Journal of *Molecular Sciences*

MDPI

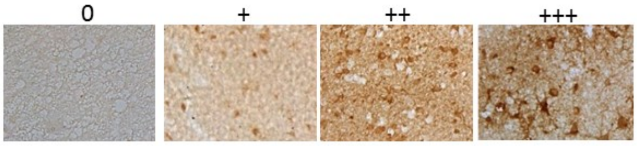

Figure S1. An indicator key for staining intensity scoring for immunohistochemical detection of PAD isozymes in protein deimination in brain sections used in this pilot study. Scoring intensity is as follows: 0 (negligible positive staining), + (low positive staining), ++ (medium positive staining intensity), +++ (strong positive staining).

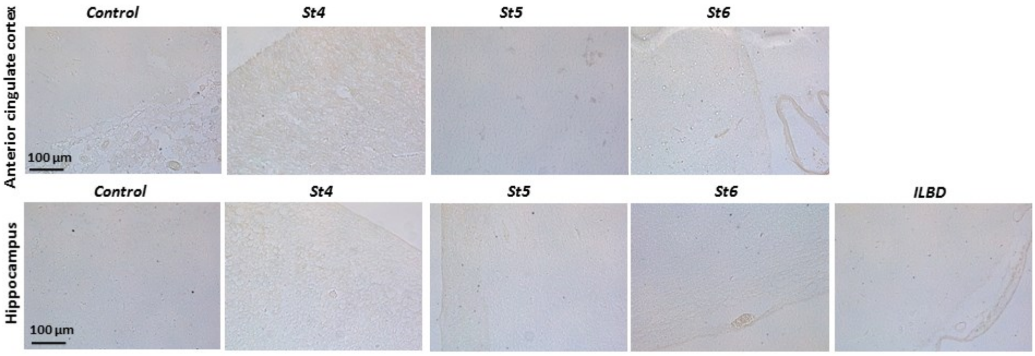

Figure S2. Negative control staining, omitting the primary antibody. Anterior cingulate cortex from control tissue alongside PD Braak stages 4, 5 and 6. Hippocampus from control tissue, from PD Braak stages 4, 5 and 6, and from incidental Lewy Body disease (ILBD). Scale bar shown (100 µm) is representative for all images.
